# Acupuncture Rescues Cognitive Impairment and Upregulates Dopamine-*β*-Hydroxylase Expression in Chronic Cerebral Hypoperfusion Rats

**DOI:** 10.1155/2018/5423961

**Published:** 2018-07-12

**Authors:** Ling-Yong Xiao, Jing-Wen Yang, Xue-Rui Wang, Yang Ye, Na-Na Yang, Chao-Qun Yan, Cun-Zhi Liu

**Affiliations:** ^1^Beijing University of Chinese Medicine, 11 Bei San Huan Dong Road, Chaoyang District, Beijing 100029, China; ^2^Department of Acupuncture and Moxibustion, Dongfang Hospital, Beijing University of Chinese Medicine, No. 6 Fangxingyuan 1st Block, Fengtai District, Beijing 100078, China; ^3^Department of Acupuncture and Moxibustion, Beijing Hospital of Traditional Chinese Medicine affiliated to Capital Medical University, 23 Meishuguanhou Street, Dongcheng District, Beijing 100010, China

## Abstract

Alteration of dopamine (DA) and noradrenaline (NA) contributes to cognitive function. Acupuncture has been shown to affect DA and NA in chronic cerebral hypoperfusion (CCH) rats. However, the effect of acupuncture on DA-*β*-hydroxylase (DBH), the biosynthetic enzyme of NA, remains unknown. In CCH rats we established chronic hypoperfusion by bilateral common carotid artery occlusion (two-vessel occlusion, 2VO) and treated them with acupuncture. Acupuncture displayed beneficial effects on hippocampus-dependent memory impairments, including nonspatial and spatial memory. That is also reflected in hippocampus long-term-potentiation (LTP). Moreover, DBH expression in the hippocampus and DBH activity in cerebrospinal fluid were upregulated after acupuncture treatment. In conclusion, these in vivo findings suggest that acupuncture exerts a therapeutic effect on hippocampus-dependent memory and hippocampus LTP in CCH rats, which may be partially related to the modulation of DBH in the hippocampus.

## 1. Introduction

When the global cerebral ischemia or hypoxia long-lasting exists, noted as chronic cerebral hypoperfusion (CCH), it predisposes to the cognitive impairment and even dementia [1]. Clinical studies evidenced that CCH is a key pathological mechanism of vascular dementia (VaD) and Alzheimer's disease (AD) [2, 3]. The persistent decreasing of cerebral blood flow (CBF) will contribute to the accumulation of beta-amyloid (A*β*) [4, 5] cell death [6] and impaired synaptic contacts [7].

Noradrenaline (NA) is related to various cognitive and physiological processes including learning and memory, dread, nervousness, arousal, mood, attention, and appetite [8]. As a key synthesizing enzyme of NA, the activity of DA-*β*-hydroxylase (DBH) influences the levels of DA and the biosynthesis of NA and epinephrine. Plasma and cerebrospinal fluid (CSF) DBH levels might be the biomarker that associates with the function of NA. Meanwhile, the inhibition or loss of noradrenergic neurons results in the decreased level of DBH in brain [9].

Acupuncture, as a nonpharmacological therapy, may be efficacious in patients with vascular dementia, one disease induced by CCH [10]. Cumulative preclinical evidence demonstrated that acupuncture elicited effects on the monoamine system, including DA, NA, and serotonin. Our recent study indicated that acupuncture at Baihui (GV20) and Zusanli (ST36) modulated the NA content in the hippocampus [11]. Given that the effect of acupuncture on DBH, the biosynthetic enzyme of NA, remains unknown, exploring this modulation may provide profound insight into acupuncture's mechanism in CCH treatment.

## 2. Materials and Methods

### 2.1. Animals and Groups

Male Wistar rats (Vital River Laboratory Animal Technology Co. Ltd., 250-300g, 7-8 weeks old) were housed under maintained temperature conditions at 22±2°C, a 12 h dark/light cycle, and humidity of 50±10%. Animals accessed water and food* ad libitum*. All procedures were sanctioned by the Ethics Committee for Animal Experimentation of the Capital Medical University and were in compliance with the NIH Guidelines for the Care and Use of Laboratory Animals. To assess acupuncture's effect on cognitive function of CCH rats, we randomly assigned rats into 5 groups: sham-operated group, bilateral common carotid artery occlusion (two-vessel occlusion, 2VO) group, 2VO + acupuncture group, 2VO + placebo-acupuncture group, and sham + acupuncture group. To explore acupuncture's effect on long-term-potentiation (LTP), rats were randomly divided into 4 groups: sham-operated group, 2VO group, 2VO + acupuncture group, and 2VO + placebo-acupuncture group.

### 2.2. Bilateral Common Carotid Arteries Occlusion

CCH was established by 2VO as previously reported [12]. Rats were anesthetized with sodium pentobarbital (40 mg/kg, i.p.). After neck ventral midline incision, bilateral common carotid arteries were exposed and separated from carotid artery sheaths and vagal nerves with caution. Then, both arteries were double ligated with a silk suture (5-0). Identical operation was performed on sham-operated rats without artery occlusion. Throughout the surgery, rectal temperature was modulated at 36.5–37.5°C with a heating pad.

### 2.3. Acupuncture and Placebo-Acupuncture Treatment

Two days after surgery, rats in acupuncture group and placebo-acupuncture group received 12 sessions of treatment, once daily, with 1 day rest every 7 days. After local barbering of the fur and disinfection, sterile acupuncture needle was inserted into the acupuncture points with a depth of 5 mm. For 2VO + acupuncture group, GV20 (sagittal midline of the head, midpoint of the line connecting the apices of the ears) [13] and bilateral ST36 (5 mm below the fibular head and 2 mm lateral to the crista anterior tibia) [13] were selected as treatment acupuncture points. For the 2VO + placebo-acupuncture group 10 mm above the anterior iliac crest was selected as the stabbing site. Everyday treatment session lasted 10 minutes. To minimize the stress, the rats were allowed to move in individual cages without anesthesia or immobilization during needle retainment.

### 2.4. Novel Object Recognition Test

**Figure 1:**
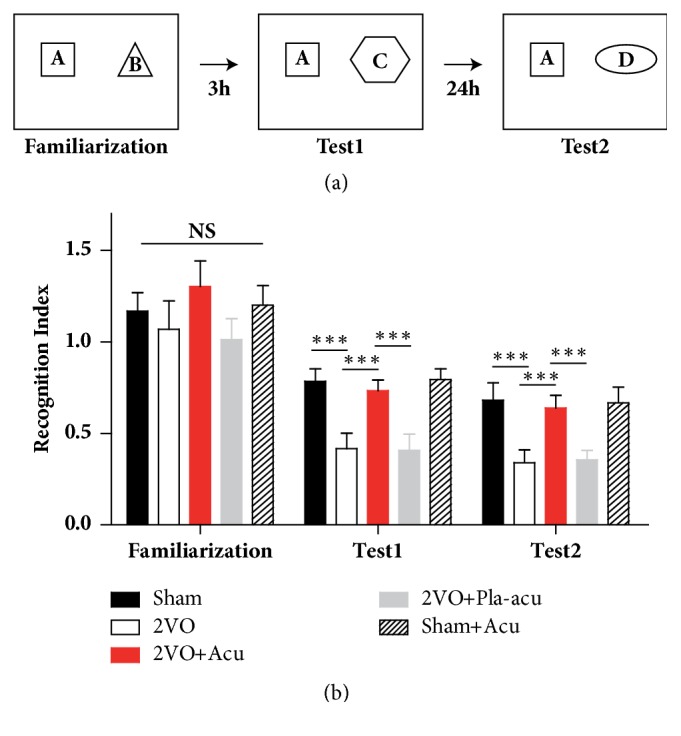
Acupuncture ameliorates episodic memory deficits in new object recognition test. (a) Illustration showing the experimental design of the novel object recognition test. Sham: sham-operated group, Acu: acupuncture group, Pla-acu: placebo-acupuncture group, Sham + Acu: sham-operated + acupuncture group. (b) The new object recognition performance of rats during the familiarization, test 1 (3 hours after familiarization), and test 2 (24 hours after familiarization). The “recognition index” for the trials was calculated as follows: recognition index = time exploring novel object/(time exploring familiar object + time exploring novel object). (n = 6 rats for each group, *∗∗∗*: P<0.001, compared as indicated.)

The novel object recognition test (NORT) protocol was based on previous studies with some slight modification to access hippocampus-mediated declarative memory [14–16]. The NORT was conducted in an opaque Plexiglas arena (80 × 40 cm^2^). Rats were acclimated to the experimental room and the empty arena on 2 consecutive days, for 30 min each time. On day 3, animals were first allowed to explore the arena containing two identical objects for 10 min and then returned to their home cages. After 3 (short-term memory) or 24 h (long-term memory), one object was displaced by a new one with a clearly distinct figure and color (test session) (Figure 1(a)). Novel objects and their positioning varied through all tests to avoid location preference. When rats sniffed or touched the object, it seemed as object exploration. Object exploration time was recorded by two experienced observers, blinded to treatment allocation. When rats reared themselves by the object or touched the object heading, another orientation was viewed as invalid exploration. The objects and arena were cleaned with ethanol following each session to prevent the influence of olfactory clue. The “recognition index” for the trials was computed according to this formula: time exploring novel object/ (time exploring familiar object + time exploring novel object). Trials were video-tracked as the NORT.

### 2.5. Radial Arm Maze

The radial arm maze (RAM) was used to assess spatial learning and memory, which has been described in previous publications with some modifications [17]. Before the RAM test, rats body weights were maintained to 80% of their body weights by controlling food intake. The maze consisted of 8 radial arms (47.5 × 14.5 × 22.5 cm) extending from the center platform (diameter = 46.5 cm). The apparatus was elevated to 70 cm above the floor. Food pellets in small metal cups were placed at the end of four arms, in such a way that the rats could not see them without completely entering that arm. The maze was placed in a room rich in salient extra maze cues for direction recognition, which were kept in the same place during the experiment. Before actual testing, rats were given 2 days of acclimation to the maze. The rats were subjected to the retrieving of the food pellets in each arm. For experimental testings, rats performed one trial per day lasting maximally 10 minutes or until the animal had collected all the pellets. Time to find all the pellets was recorded. Consumption of each pellet was recorded as the correct choice. Omission error was defined as visiting an arm without food consumption, as an indication of motivation. Working memory error was defined as entries into arms which had been visited previously during a trial. A overhead camera above the maze was used to record the sessions. Video tracking software (TopScan Lite, Clever Sys. Inc.) was used to quantify animal's performance throughout all sessions.

### 2.6. Electrophysiological Recordings In Vivo

Electrophysiological recordings in the dentate gyrus (DG) were carried out as described previously [18]. Once the treatment ended, rats were anesthetized with urethane (1.2 g/kg, i.p; supplemental doses of 0.3–0.6 g/kg s.c. as necessary) [19], and the body temperature was maintained at 37°C with a heating pad. All recordings were made in a blinded manner to group assignment. Two small holes were drilled onto the right side of the parietal bone for stimulating electrode (perforant path, PP, AP -8 mm; L 4.4 mm; V 3.0–3.5 mm) and recording electrode (DG, AP -3.8 mm, L 2.2 mm, V 3.0-3.5 mm) respectively. The reference electrode was placed over the neck area. A test stimulation (60 *μ*s in duration, 250-400 *μ*A in intensity) was applied, with an interval of 60 s. The final location of recording and stimulating electrodes was refined according to the amplitude of evoked potentials by test stimulation. Subsequent to 20 min steady baseline recording, tetanic stimulation of 4 trains of 30 stimuli at 400 HZ with 10 s intertrain interval was applied and the response was recorded, amplified, and analyzed with CED Spike 2 signal processing system.

### 2.7. Western Blotting

After protein concentrations determination with Pierce BCA Protein Assay kit (Thermo Scientific), the hippocampus homogenate was separated on 12% SDS-PAGE (Applygen) and electrically transferred onto polyvinylidene fluoride membranes. Subsequent to blocking by 5% nonfat milk for 1 hour, the membranes were incubated overnight at 4°C with DBH Antibody (1:200, sc-47707, Santa Cruz) or GAPDH (1:1000, Bioss). On the 2^nd^ day, the membranes were incubated with horseradish peroxidase-conjugated secondary antibodies for 1 hour at room temperature. Immunoblots were scanned using the Odyssey Infrared Imaging System (LI-COR Biosciences, Nebraska, USA).

### 2.8. Cerebrospinal Fluid (CSF) Collection

The procedure of CSF collecting was based on previous study [20] and modified as follows: rats were anesthetized with sodium pentobarbital (40 mg/kg, i.p.). After local shaving of the neck, the anesthetized rat was mounted in a stereotaxic apparatus and fixed. The angle between animal's head and horizontal line was maintained at 45°. Disinfection by ethanol made the pit between the occipital protuberance and the spinal process C1 distinct. A needle attached to a syringe was inserted gently into the cisterna magna for CSF extraction without making incision at this area. Once resistance feeling disappeared, the colorless CSF was gently extracted by controlling the syringe. Upon observing the blood contamination, the PE tube was bended to prevent the contamination sample from being aspirated into the syringe. Then the tube was cut and the contaminated sample was discarded. The collected colorless CSF was stored at -80°C.

### 2.9. Plasma Collection

After CSF collection, the rats were then subjected to plasma collection. After sternotomy incision and heart exposure, approximately 5 ml of cardiac blood was extracted in heparin-coated tubes on ice. Then the blood was subjected to centrifugation at 1600 g for 15 min at 4°C to get the plasma. The plasma was kept at -80°C before activity assay.

### 2.10. Enzyme-Linked Immunosorbent Assay (ELISA)

DBH activity in the cerebrospinal fluid and plasma was measured by an ELISA kit (JONLN, JL20967-48T) according to the manufacturer's protocol. Absorbance at *λ*_450_ nm in each well was measured by a microplate reader (Thermo Fisher, USA). The levels of DBH were expressed as pg/ml.

### 2.11. Statistical Analysis

The analyses were conducted using SPSS software version 20.0, and *α*=0.05 was set for statistical significance. All data were expressed as mean ± SEM. Two-way repeated measure ANOVA was used to analyze the results of NORT and RAM. One-way ANOVA followed by Tukey's test was adopted to analyze the results of LTP, Western blotting, and ELISA.

## 3. Results

### 3.1. Effects of Acupuncture on Episodic Memory Deficits Induced by 2VO

We firstly conducted NORT, which is considered to be the mammalian declarative memory [14]. As shown in Figure 1(b), during the familiarization phase, there was no difference in time spent by the five groups on the identical objects. During test 1 and test 2, only treatment had significant effects (Figure 1(b), time: P > 0.05; treatment: P < 0.001; interaction of time and treatment: P>0.05). 2VO rats did not spend more time investigating the novel object, as compared to the sham-operated rats (Figure 1(b), sham-operated versus 2VO: P < 0.001). In contrast, rats treated by acupuncture explored the novel object for longer time than 2VO rats (Figure 1(b), 2VO + acupuncture versus 2VO: P < 0.05). The performance of rats treated by placebo-acupuncture was identical to that of 2VO rats (Figure 1(b), 2VO + placebo-acupuncture versus 2VO: P > 0.05). In addition, the difference between the sham-operated and sham + acupuncture groups was not significant (Figure 1(b), sham-operated versus sham + acupuncture: P > 0.05).

### 3.2. Acupuncture's Effect on Spatial Memory Deficits Induced by 2VO

**Figure 2:**
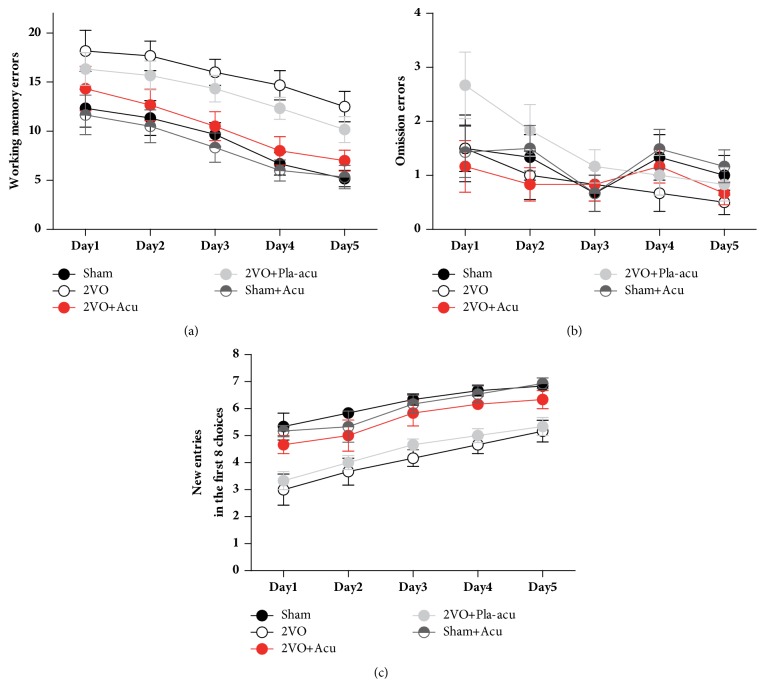
Acupuncture ameliorates spatial memory deficits in radial arm maze test. Sham: sham-operated group, 2VO + Acu: 2VO + acupuncture group, 2VO + Pla-acu: 2VO + placebo-acupuncture group, Sham + Acu: sham-operated + acupuncture group. (a) Total number of working memory errors across training. (b) The number of new entries (i.e., different arms chosen) in the first eight arm visits. (c) Number of omission errors, i.e., arm was visited but food was not taken, as an indication of motivation. (n = 6 rats for each group.)

To determine whether acupuncture can rescue the impaired hippocampus-dependent memory deficits caused by CCH, we then evaluated spatial learning and memory using the RAM test. For working memory errors, both the time and treatment had significant effects (Figure 2(a), time: P < 0.001; treatment: P < 0.05; interaction of time and treatment: P > 0.05). The results showed that the average number of working memory errors was significantly higher for the 2VO rats than that for the sham-operated rats (Figure 2(a), sham-operated versus 2VO: P < 0.05), indicating a spatial memory deficit after CCH. However, rats treated by acupuncture displayed fewer errors than 2VO rats (Figure 2(a), 2VO + acupuncture versus 2VO: P < 0.05). Intriguingly, the errors of rats receiving placebo-acupuncture were comparable to those of 2VO rats (Figure 2(a), 2VO + placebo-acupuncture versus 2VO: P > 0.05) and significantly higher than those of rats treated by acupuncture (Figure 2(a), 2VO + placebo-acupuncture versus 2VO + acupuncture: P < 0.05). No significant difference between sham-operated rats and sham + acupuncture rats was found (Figure 2(a), sham-operated rats versus sham + acupuncture: P > 0.05).

The five groups made similar numbers of omission errors (Figure 2(b), P > 0.05), demonstrating that the 2VO rats did not have remarkable motivational impairment and acupuncture did not elicit effects on the motivation of eating.

The number of new entries in the first 8 choices is another measure of spatial memory performance. For this measure, there was a significant effect for time and treatment, respectively. The treatment × time interaction was not significant (Figure 2(c), time: P < 0.001; treatment: P < 0.001; interaction of time and treatment: P > 0.05). The sham-operated rats exhibited good spatial memory by visiting markedly more different arms, whereas 2VO rats showed less new arms choices (Figure 2(b), sham-operated versus 2VO: P < 0.001). After treatment with acupuncture, the number of new entries increased (Figure 2(b), 2VO + acupuncture versus 2VO: P < 0.001). Similar to the results of working memory errors, the placebo-acupuncture did not significantly affect the number of new entries (Figure 2(b), 2VO + placebo-acupuncture versus 2VO: P > 0.05). There was no difference between Sham rats and Sham + Acu rats (Figure 2(c), sham-operated rats versus sham + acupuncture: P > 0.05).

Overall, these results showed that acupuncture, not placebo-acupuncture, ameliorated the hippocampus-dependent memory deficits induced by CCH.

### 3.3. Effects of Acupuncture on LTP Deficits Induced by 2VO

**Figure 3:**
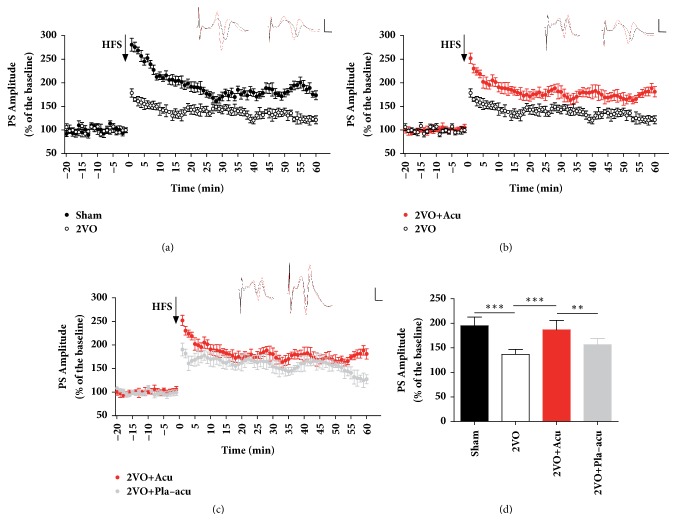
Acupuncture rescues long-term-potentiation deficits induced by 2VO. Sham: sham-operated group, 2VO + Acu: 2VO + acupuncture group, 2VO + Pla-acu: 2VO + placebo-acupuncture group. (a) 2VO (colorless circles) decreased long-term-potentiation (LTP) induced by high frequency stimulation (HFS, arrow) in the perforant path-dentate gyrus (PP-DG) area versus those of Sham rats (black circles). (b) Acupuncture (red circles) increased PP-DG LTP induced by HFS versus those of 2VO rats. (c) Placebo-acupuncture (gray circles) did not enhance LTP of VD rats. (d) The histogram showing the level of LTP during 60 min after HFS. (n = 6 rats for each group.) (*∗∗*: P<0.05,*∗∗∗*: P<0.001, compared as indicated.) Inset traces are sample traces recorded before (black) and after (red) HFS. Horizontal bar: 5 ms; vertical bar: 5 mV.

To determine whether acupuncture can rescue the impaired synaptic plasticity caused by 2VO, LTP of population spike (PS) was recorded in DG of the hippocampus. 2VO rats exhibited impaired LTP compared to the sham-operated rats (Figures 3(a) and 3(d), sham-operated (194.9% ± 17.9) versus 2VO (136.6% ± 10.3): P < 0.001). Animals treated by acupuncture showed elevated PS compared to 2VO rats (Figures 3(b) and 3(d), 2VO + acupuncture (186.7% ± 19.3) versus 2VO: P < 0.001), whereas PS was unaffected by placebo-acupuncture (Figures 3(c) and 3(d), 2VO + placebo-acupuncture (156.4% ± 12.8) versus 2VO: P > 0.05).

### 3.4. Effects of Acupuncture on DBH Expression in Hippocampus

**Figure 4:**
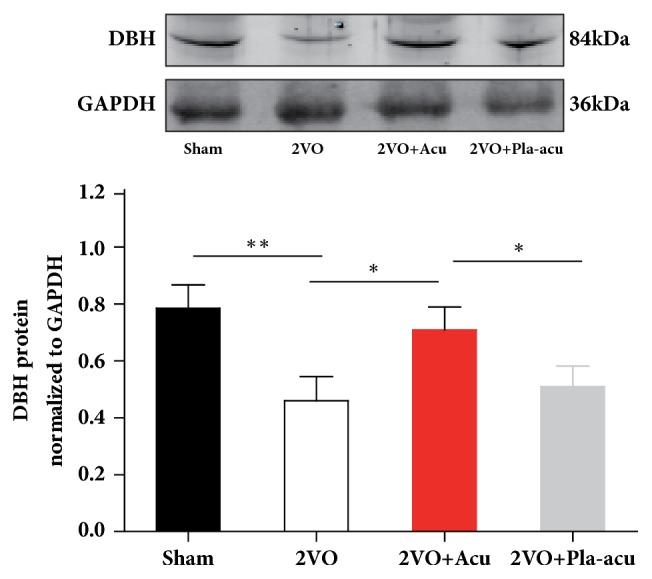
Acupuncture elevates the expression of dopamine-*β*-hydroxylase in hippocampus induced by 2VO. Sham: sham-operated group, 2VO + Acu: 2VO + acupuncture group, 2VO + Pla-acu: 2VO + placebo-acupuncture group. Values are expressed as means ± SEM. (n = 6 rats for each group.) (*∗*: P<0.05, *∗∗*: P<0.05, compared as indicated.)

To determine whether acupuncture affects expressions of DBH, we measured protein levels of DBH in hippocampus. The expression of DBH was decreased by 2VO in regard to the sham-operated rats (Figure 4, sham-operated versus 2VO: P < 0.05). Treatment with acupuncture leaded to elevated expression of DBH compared to 2VO rats (Figure 4, 2VO + acupuncture versus 2VO: P < 0.05). The placebo-acupuncture did not elevate the DBH expression (Figure 4, 2VO + placebo-acupuncture versus 2VO: P>0.05).

### 3.5. Effects of Acupuncture on DBH Activity in CSF and Plasma

**Figure 5:**
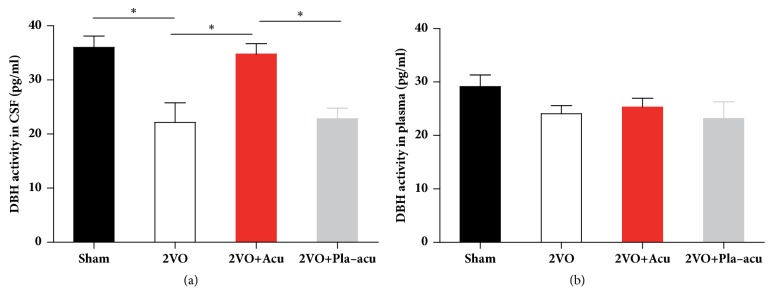
Acupuncture increases dopamine-*β*-hydroxylase activity in CSF but not in plasma. Sham: sham-operated group, 2VO + Acu: 2VO + acupuncture group, 2VO + Pla-acu: 2VO + placebo-acupuncture group. Values are expressed as means ± SEM (n = 6 rats for each group.) (*∗*: P<0.05 compared as indicated.)

DBH activity in CSF of 2VO rats was lower than that of the sham-operated rats (Figure 5(a) sham-operated versus 2VO: P < 0.05). After treatment with acupuncture, there was a significant rise of DBH activity in CSF (Figure 5(a), 2VO + acupuncture versus 2VO: P < 0.05). Conversely, placebo-acupuncture had no significant effect on DBH activity in CSF compared with 2VO rats (Figure 5(a), 2VO + placebo-acupuncture versus 2VO: P > 0.05).

In regard to DBH activity in plasma, there was no significant difference among the 4 groups (Figure 5(b) P > 0.05).

## 4. Discussion

This study demonstrated that acupuncture attenuated hippocampus-dependent memory deficits and LTP impairment induced by 2VO. In parallel with that, decreased DBH levels were also elevated by acupuncture. Thus, the improvement of cognitive function and synaptic plasticity by acupuncture may be at least partially related to DBH in the hippocampus.

As demonstrated in previous preclinical studies, cerebral ischemia or hypoperfusion contributes to cognitive impairment [21–24]. The present study is consistent with these results. The 2VO is a classical animal model and used to study the effects of CCH on synaptic plasticity and memory performance. A number of preclinical studies indicated that acupuncture improves cognitive deficits induced by 2VO, including spatial and nonspatial memory. We used RAM to assess the spatial memory of animals, which avoids the stress of imposing swimming. In addition to spatial memory, declarative memory is another cognitive dysfunction seen in CCH patients [25]. Thus, the animals underwent the NORT to determine declarative memory deficits.

The selection of acupoints including GV20 and ST36 is based on previous study which suggested that the effects elicited by the combination of GV20 and ST36 on spatial learning and memory were superior over other acupoints combination [18]. Neuroprotective effect of GV20+ST36 on CCH has been demonstrated in several preclinical studies [18, 26–28] which were consistent with the current study. Although the combination GV20+ST36 is one of the most frequent combinations for VaD treatment [29], high quality clinical trials are needed to further demonstrate the clinical effect of the combination [30].

The field excitatory postsynaptic potential (fEPSP) slope is used to assess synaptic efficacy [19, 31] and our previous study demonstrated an enhancing effects of acupuncture on fEPSP in hippocampus [18]. The PS amplitude reflects the number and harmony of exciting granule cells [32]. In addition to synaptic efficiency, PS may also suggest activity of the soma of the neurons [31]. The present study showed that acupuncture elevated the PS, which indicates acupuncture may increase the number and prompt the synchrony of firing GCs in DG.

It has been reported that a putatively functional single nucleotide polymorphism in the DBH, NA biosynthetic enzyme, is associated with cognitive function [33]. DBH knock-out mice displayed specific memory deficits [34, 35]. A study of our group showed that catecholamine in the hippocampus could be increased by acupuncture [18]. The upregulated DBH may subsequently convert DA to NA and further affects cognitive function and plasticity in hippocampus.

It is commonly believed that the induction and maintenance of hippocampal LTP constitute a good indicator for memory performance [36]. In addition to that, they are also considered as one important mechanism underlying memory [37]. Release and roles of neurotransmitter at the synapse area are related to synaptic activity [38]. We have demonstrated previously that the content of neurotransmitters, including DA and NA, could be elevated by acupuncture [11, 18]. The responding receptor localized in postsynaptic membrane could also be upregulated by acupuncture [11, 18]. Thus, synaptic plasticity related to the improvement of cognitive function may involve both presynaptic and postsynaptic mechanism.

Limitations of this study should be noted. First, the current study presents the pathological changes of 2 weeks after 2VO operation, while the pathological process of CCH is dynamic. Second, we focused on a single protein in cognitive dysfunction while the dysfunction is likely to be joint action of more than one protein. Comprehensive understanding of the dynamic interaction of DA, DBH, NE, and other noradrenergic components at different disease stages may contribute to the full demonstrating of acupuncture's mechanism of memory improvement in CCH.

## 5. Conclusion

In conclusion, these data evidenced that acupuncture treatment improved hippocampus-dependent memory and synaptic plasticity in CCH. This improvement may be related to the modulation of DBH in the hippocampus.
